# NLR diversity and candidate fusiform rust resistance genes in loblolly pine

**DOI:** 10.1093/g3journal/jkab421

**Published:** 2021-12-13

**Authors:** Daniel Ence, Katherine E Smith, Shenghua Fan, Leandro Gomide Neves, Robin Paul, Jill Wegrzyn, Gary F Peter, Matias Kirst, Jeremy Brawner, C Dana Nelson, John M Davis

**Affiliations:** 1 School of Forest, Fisheries, and Geomatics Sciences, University of Florida, Gainesville, FL 32611, USA; 2 USDA Forest Service, Southern Research, Southern Institute of Forest Genetics, Saucier, MS 39574, USA; 3 Forest Health Research and Education Center, University of Kentucky, Lexington, KY 40546, USA; 4 Department of Horticulture, University of Kentucky, Lexington, KY 40546, USA; 5 Rapid Genomics, Gainesville, FL 32601, USA; 6 Department of Ecology and Evolutionary Biology, University of Connecticut, Storrs, CT 06269, USA; 7 Department of Plant Pathology, University of Florida, Gainesville, FL 32611, USA; 8 USDA Forest Service, Southern Research Station, Forest Health Research and Education Center, Lexington, KY 40546, USA

**Keywords:** NLR, RNAseq, fusiform rust, resistance genes, *Pinus taeda*, Fr genes, sequence-capture, pan-NLRome

## Abstract

Resistance to fusiform rust disease in loblolly pine (*Pinus taeda*) is a classic gene-for-gene system. Early resistance gene mapping in the *P*. *taeda* family 10-5 identified RAPD markers for a major fusiform rust resistance gene, *Fr1*. More recently, single nucleotide polymorphism (SNP) markers associated with resistance were mapped to a full-length gene model in the loblolly pine genome encoding for a nucleotide-binding site leucine-rich repeat (NLR) protein. NLR genes are one of the most abundant gene families in plant genomes and are involved in effector-triggered immunity. Inter- and intraspecies studies of NLR gene diversity and expression have resulted in improved disease resistance. To characterize NLR gene diversity and discover potential resistance genes, we assembled de novo transcriptomes from 92 loblolly genotypes from across the natural range of the species. In these transcriptomes, we identified novel NLR transcripts that are not present in the loblolly pine reference genome and found significant geographic diversity of NLR genes providing evidence of gene family evolution. We designed capture probes for these NLRs to identify and map SNPs that stably cosegregate with resistance to the SC20-21 isolate of *Cronartium quercuum* f.sp. *fusiforme* (Cqf) in half-sib progeny of the 10-5 family. We identified 10 SNPs and 2 quantitative trait loci associated with resistance to SC20-21 Cqf. The geographic diversity of NLR genes provides evidence of NLR gene family evolution in loblolly pine. The SNPs associated with rust resistance provide a resource to enhance breeding and deployment of resistant pine seedlings.

## Introduction

### Plant NLR proteins

In plant species, disease resistance genes (R genes) often encode nucleotide-binding site leucine-rich repeat (NLR) proteins, a large family of immune receptors characterized by an N-terminal domain, a nucleotide-binding site, and C-terminal leucine-rich repeat domains (Jones and Dangl 2006). NLR proteins are intracellular immune receptor proteins and detect the invasion of the host by insects and pathogens (van der Hoorn and Kamoun 2008; Cesari *et al.* 2014; Tamborski and Krasileva 2020). NLR proteins play key roles in disease resistance to biotrophic pathogens, where disease is typically governed by the gene-for-gene model in which symptom expression is conditioned by pathotype-specific genetic interactions between R gene alleles and pathogen genotypes harboring specific (a)virulence alleles (Flor 1971; Bent *et al.* 1994; Mindrinos *et al.* 1994; Whitham *et al.* 1994; Botella *et al.* 1996; Ellis *et al.* 2000). Given the important role of NLR genes in regulating disease resistance, gene family members were identified and their diversity characterized in model and crop plant species (Van Ghelder *et al.* 2019; Van de Weyer *et al.* 2019; Barragan and Weigel 2020; Scott *et al.* 2020).

Recent efforts to sequence and assemble the genomes of ecologically and economically important conifer species were driven in part by their vulnerability to native and introduced pathogens (Neale *et al.* 2014; Wegrzyn *et al.* 2014; Stevens *et al.* 2016; Van Ghelder *et al.* 2019; Scott *et al.* 2020). Conifer genomes contain a large repertoires of NLRs. Along with their traditional role as disease proteins, studies of NLR gene expression in conifers indicate they may also play a role in response to abiotic stress (Van Ghelder *et al.* 2019). For example, sequencing and annotating the massive sequoia genome revealed over 900 complete or partial predicted NLR genes, with over one-third of them supported by expression evidence (Scott *et al.* 2020). The loblolly and sugar pine genomes were shown to harbor numerous NLR genes, and individual genes were shown to associate with resistance to biotrophic pathogens (Neale *et al.* 2014; Wegrzyn *et al.* 2014; Stevens *et al.* 2016). The large cohort of NLR genes in conifers motivates further research to identify NLR genes that impact biotic and abiotic stress tolerance.

### Resistance to fusiform rust

Resistance to fusiform rust in *Pinus* *taeda* follows a “gene-for-gene” interaction model between the host and the pathogen, *Cronartium quercuum* f.sp. *fusiforme* (Cqf) (Wilcox *et al.* 1996; Amerson *et al.* 1997; Stelzer *et al.* 1999; Nelson *et al.* 2010). Progeny from trees that reliably segregate for resistance when inoculated with single-spore pathogen isolates in controlled disease screening studies have been used to locate R genes (Zobel and Talbert 1984; Wilcox *et al.* 1996; Nelson *et al.* 2010). Trees are inoculated as eight-week-old seedlings and 24 weeks later, they are scored for stem gall presence or absence. The first fusiform rust resistance gene was designated “*Fr1*” (Kuhlman and Powers 1988; Wilcox *et al.* 1996). *Fr1* was found to segregate in the progeny of the loblolly family designated “10-5.” This family has been used extensively to map R genes (Wilcox *et al.* 1996; Nelson *et al.* 2010; Quesada *et al.* 2014; Amerson *et al.* 2015).

An analysis of the interactions between five single-spore pathogen isolates and seven loblolly pine families identified a total of nine *Fr* genes that were consistently organized as clusters on four linkage-groups in two linkage-maps (Amerson *et al.* 2015). This genomic organization of fusiform rust R genes in loblolly pine is consistent with findings from model and crop plant genomes where NLR genes are organized in genomic clusters (Michelmore and Meyers 1998; Meyers *et al.* 2003). Intra-cluster recombination and gene conversion are thought to generate diversity within these clusters (Michelmore and Meyers 1998; Noël *et al.* 1999; Meyers *et al.* 2003; Kuang *et al.* 2004; Barragan and Weigel 2020).

Later studies placed a single nucleotide polymorphism (SNP) associated with the *Fr1* resistance gene in a full-length NLR gene model in the assembled *P*. *taeda* genome (Neale *et al.* 2014). Importantly, the avirulence locus in the pathogen that specifically interacts with *Fr1* (*Avr1*) was identified in the genetic map of Cqf by bulked segregant mapping in a population segregating for avirulence to *Fr1* (Kubisiak *et al.* 2005, 2011). This conclusively demonstrated a classical gene-for-gene interaction model (Flor 1971). Given the gene-for-gene architecture of fusiform rust disease resistance in loblolly pine, and the abundance of NLR genes in conifers, we used RNA sequencing (RNAseq) to discover novel NLR genes in the transcriptomes of highly resistant pine families. Additionally, we conducted linkage-mapping and a genome-wide association study (GWAS) within a pine family 10-5 known to segregate for the *Fr1* resistance gene using a set of sequence-capture probes designed to target sequences on the linkage group believed to harbor the *Fr1* resistance gene, genome wide markers in the loblolly pine genome, and novel NLR transcripts discovered in our transcriptomic dataset.

## Materials and methods

### Sample selection and approach

To discover novel NLR-encoding transcripts, we sequenced the transcriptomes of 92 unrelated maternal half-sib families of loblolly pine. The families represented five seed sources (provenances) distributed across the natural range of loblolly pine, including Arkansas (AR), Texas (TX), and Louisiana/Mississippi (LA/MS) in the west as well as Piedmont (PDMT) and Atlantic Coastal Plain (ACP) sources in the east. They were chosen based on their maternal parent’s importance to industry breeding programs including high levels of fusiform rust resistance. RNA was extracted from stem tissue collected from 10 open-pollinated (OP) seedlings (8 weeks from germination, see details below) from the same family to make a single-family pool. The 92 single-family tissues were combined into 30 pools for RNA extraction and barcoded for library construction (Supplementary Table S1). Eighteen contained RNA from between three and seven families per pool, while 12 contained RNA from a single family. The 12 families (4 PDMT and 8 ACP) sequenced as single-families were among the most rust-resistant families identified in industrial breeding programs.

Using sequence capture, we genotyped 291 OP seedlings from a single parent (10-5) that is known to be heterozygous for the *Fr1* resistance allele (Wilcox *et al.* 1996; Nelson *et al.* 2010; Amerson *et al.* 2015). In addition, to facilitate mapping of *Fr1* in this family, we genotyped 32 samples of haploid tissue from megagametophytes dissected from OP 10-5 seeds, a diploid sample of loblolly pine 20-1010 (the tree that provided the DNA for the loblolly pine genome reference sequence), and DNA from four families used to generate the transcriptome (two from the PDMT source and two from the ACP source). Both 10-5 and 20-1010 originated from the ACP.

### RNAseq library construction, sequencing, and transcriptome mining

**Figure 1:**
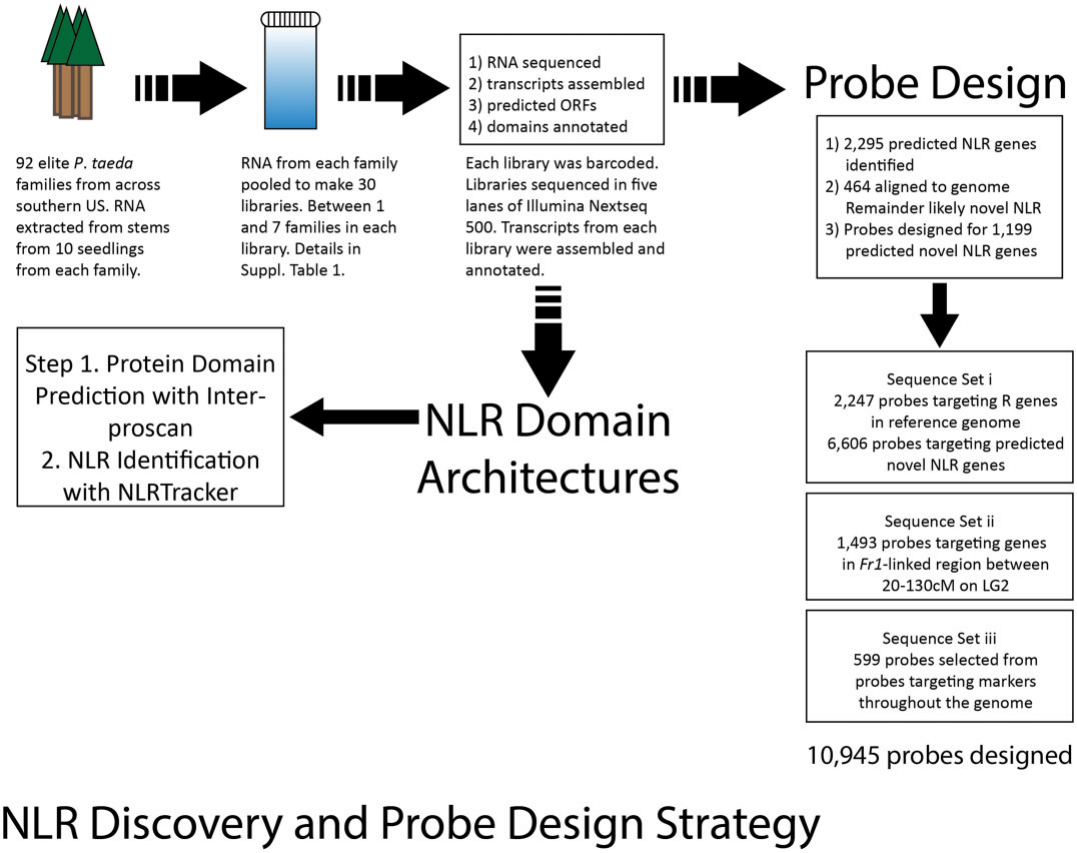
NLR gene discovery and probe design strategy.

From each of ten 8-week-old greenhouse-grown seedlings, 5–7 cm of succulent epicotyl tissue was harvested and the needles were removed. The stem tissues were pooled by family as they were collected. The stems were flash frozen using liquid nitrogen and transferred to a –80°C freezer and then freeze-dried prior to RNA extraction. Tissue was ground using a MiniG^®^ tissue homogenizer (SPEX Sample Prep, Metuchen, NJ) and 5/32-inch stainless steel balls. RNA was extracted using the RNAqueous^®^-Micro Total RNA Isolation Kit (Ambion) and DNA was removed using the TURBO DNA-free™ kit (Ambion). RNA pools were constructed such that each family represented in a pool contributed an equal amount of RNA and the total amount of RNA for each pool equaled 1 ug (Supplementary Table S1, Figure  1), and then libraries were prepared from each pool using the NEBNext^®^ Ultra™ RNA Library Prep Kit for Illumina^®^. The 30 libraries were sequenced in four lanes of an Illumina NextSeq 500, with 150 cycles and paired-end format. The 150 base paired-end reads for each library were trimmed and filtered for quality and length with Sickle (minimum Q = 30, minimum length = 45 bp) (Joshi and Fass 2011). For each library, transcripts were assembled with Trinity (300 bp minimum) and open-reading frames were identified in the transcripts with Transdecoder (Haas *et al.* 2013).

### Annotation of NLR domain architectures

Protein domains were annotated with CATH-Gene3D, SUPERFAMILY, PRINTS, PROSITE, SMART, CDD, and Pfam using InterProScan, and predefined NLR-motifs were annotated using the meme-suite following the methods developed for the RefPlantNLR database (Bailey *et al.* 2009; Jupe *et al.* 2012; Kourelis *et al.* 2020). NLR protein domain architectures were identified with the NLRTracker tool as described in (Kourelis *et al.* 2020).

### Sequence-capture probe design

Since there was evidence that an *Fr1* candidate gene encodes a TIR-NLR protein (Neale *et al.* 2014), TIR-NLR encoding transcripts were identified by searching the translated coding-sequences against the Pfam and SMART domain/motif databases with InterProScan (Letunic *et al.* 2015; Finn *et al.* 2016; Letunic and Bork 2018; El-Gebali *et al.* 2019). Transcripts with characteristic TIR-NLR domains (either NB-ARC or TIR domains in conjunction with an NBS domain and/or LRR domain) were selected for further analysis and clustered with uclust (Li and Godzik 2006; Fu *et al.* 2012). Putative TIR-NLR genes were then aligned to the *P.* *taeda* v1.01 reference genome assembly (“Pita v1.01 genome”) with gmap (Neale *et al.* 2014; Wegrzyn *et al.* 2014; Wu *et al.* 2016) prior to exon selection.

Hybridization probes were designed to capture three complementary sets of sequences: (1) putative TIR-NLR genes identified in pooled transcriptomes of the elite rust resistant pine families described above and in the Pita v1.01 genome, (2) genes on Pita v1.01 genome scaffolds that mapped to linkage group 2 (LG2 contains *Fr1*), and (3) genes randomly distributed throughout the genome (Supplementary Figure S1). For set (1), we designed 2247 probes to enrich NLR genes in the Pita v1.01 genome and 6606 probes to enrich NLR genes in the transcriptome data. For set (2), to select probes linked to *Fr1* on LG2, we first obtained the Pita v1.01 scaffolds corresponding to markers that were previously mapped near *Fr1* (Neale *et al.* 2014; Quesada *et al.* 2014; Amerson *et al.* 2015) and identified their position on a consensus genetic map for loblolly pine (Westbrook *et al.* 2015). All genetically mapped scaffolds between positions 20–130 cM on (LG2) from the consensus map were used to design 1493 probes for genes annotated within the scaffolds. For set (3), a total of 599 probes were randomly selected from an optimized and validated probe set (Neves *et al.* 2014), representing an average of 50 probes per linkage group. In total, we designed and synthesized 10,945 probes as previously described (Neves *et al.* 2014) for exome capture and subsequent sequencing.

### Family 10-5 and rust resistance screening with a single-spore isolate

Open pollinated (OP) seeds were collected from a ramet of 10-5 (*Fr1*/*fr1*) that was grafted into a clonal seed orchard that is managed for seed production by Arborgen Inc. The OP seedlings were inoculated at the Resistance Screening Center in Asheville, North Carolina using modification of a protocol developed by the US Forest Service for large-scale rust resistance screening (Anderson *et al.* 1982; Walkinshaw and Anderson 1988; Cowling and Young 2013; Young *et al.* 2018). We used basidiospores from a single uredinial pustule (SUP) of an isolate known to be avirulent to *Fr1* (SC20-21), to inoculate the pine seedlings instead of a mixture of basidiospores cultured from several aeciospore collections (Amerson *et al.* 2015). Prior to initiating this study, as part of the SUP protocol, urediniospores from isolate SC20-21 were genotyped with SSR markers to ensure it was not contaminated with other isolates (Burdine *et al.* 2007; Kubisiak *et al.* 2011). SC20-21 is avirulent to *Fr1* (*Avr1*/*Avr1*), and therefore does not incite galls on *Fr1*/– trees and does incite galls on *fr1*/*fr1* trees (Kuhlman and Matthews 1993; Kuhlman *et al.* 1997; Kubisiak *et al.* 2011; Amerson *et al.* 2015).

Seedlings were hedged to produce multiple shoots as a means to increase potential infection sites. Following hedging, 874 seedlings were inoculated with a spore concentration of 20,460 sp/mL.

The hedged and inoculated seedlings were scored as galled if any shoot on the seedling generated a gall and nongalled if no shoots on the seedling generated galls. At 6-months post inoculation, galls were observed on 225 out of the 874 seedlings. Since SC20-21 does not incite galls on *Fr1*/– trees and does incite galls on *fr1*/*fr1* trees (Supplementary Figure S1), we selected approximately equal numbers of galled (148) and nongalled (143) seedlings for targeted genome resequencing (Table  1) to search for markers linked to *Fr1*.

**Table 1:** Genome sequence capture sample summary

Sample type	Number of samples	Tissue sampled for DNA	Expected status at *Fr1* locus	Sample description
10-5 Mega.	32	Individual megagametophytes	Each is either *Fr1* or *fr1*	Individual haploid samples
10-5 OP ungalled	144	Individual seedlings (leaf)	*Fr1* frequent	Resistant seedlings and escape seedlings
10-5 OP galled	148	Individual seedlings (leaf)	*Fr1* rare	Susceptible seedlings
10-5 × 4-6664	6	Individual seedlings (leaf)	*Fr1* frequent	Full-sib samples from a prior 10-5 cross
20-1010	1	Individual (leaf)	Unknown	Source for reference genome assembly
CL04	1	Tissue level pool of 10 individuals (stem)	Unknown	Elite rust-resistant family, ACP source
CL05	1	Tissue level pool of 10 individuals (stem)	Unknown	Elite rust-resistant family, ACP source
PD18	1	Tissue level pool of 10 individuals (stem)	Unknown	Elite rust-resistant family, PDMT source
PD35	1	Tissue level pool of 10 individuals (stem)	Unknown	Elite rust-resistant family, PDMT source

### Sample selection for targeted genome resequencing

In addition to the 291 phenotyped seedlings of the 10-5 family that were sequenced, samples from other sources were included (Table  1). Six trees from a 10-5 (*Fr1/fr1*) × 4-6664 (*fr1*/*fr1*) full-sib family maintained as grafted trees at the Harrison Experimental Forest (Saucier, MS) were included to expand the number of 10-5 related samples (Kuhlman 1992; Wilcox *et al.* 1996). A sample tree from genotype 20-1010 (the same tree used for the Pita reference genomes) was also included (Neale *et al.* 2014; Wegrzyn *et al.* 2014; Zimin *et al.* 2017). Four libraries were prepared from single-family pools of DNA from 10 individuals from ACP and PDMT families: CL04, CL05, PD18, and PD35. The original 10-5 tree was selected from Jasper County, South Carolina in 1958, and thus falls into the ACP source (personal communication, NCSU Tree Improvement Program; Wilcox *et al.* 1996).

### DNA extraction, target enrichment, and sequencing

All samples were freeze-dried prior to DNA extraction except for megagametophytes, which were excised from pine seeds and ground fresh. Samples were ground using a MiniG^®^ tissue homogenizer (SPEX Sample Prep, Metuchen, NJ) and 5/32-inch stainless steel balls. DNA was extracted from all samples using the NucleoSpin^®^96 Plant II kit (Macherey-Nagel).

DNA was submitted to RAPiD Genomics (Gainesville, FL, USA), for library construction, target enrichment, and sequencing, following protocols previously described for loblolly pine (Neves *et al.* 2013). Briefly, an average of 500 ng of DNA was sheared to an average fragment length of 300–500 bp, end-repaired and ligated to Illumina TruSeq compatible adapters containing unique indexes to identify the samples upon sequencing. Properly ligated libraries were enriched by PCR and hybridized to the probes following Agilent’s SureSelect protocol. A total of 334 target-enriched libraries were then sequenced on an Illumina HiSeq 3000 machine using a paired-end 150 bp cycle.

### Read Quality Control (QC), alignment, variant-calling, and variant QC

The read-pairs were trimmed with cutadapt: 10 bases were trimmed from the 5′ and 3′ ends of each read, and reads were trimmed for quality by removing bases with a quality lower than 30 from the 5′ and 3′ ends of each read. Trimmed reads shorter than 50 bases were discarded.

The trimmed reads were aligned to the loci targeted by the 10,945 hybridization probes (2030 genomic scaffolds and 1199 novel NLR gene transcripts) with bwa-mem with default parameters (Li and Durbin 2009). The aligned reads (bams) were sorted and duplicated reads removed with samtools (Li *et al.* 2009). The bams from each lane were merged with picard MergeSamFiles and read group I.D.s were replaced with picard AddOrReplaceReadGroups (Picard toolkit 2019).

Insertion–deletion regions were identified with GATK RealignerTargetCreator and reads within insertion–deletion regions were realigned with GATK IndelRealigner. Variants were identified in each sample individually with GATK HaplotypeCaller, and the resulting gVCF files were combined with GATK CombineGVCFs (McKenna *et al.* 2010; Van der Auwera *et al.* 2013; Poplin *et al.* 2018; Van der Auwera and D O’Connor 2020). Joint-genotypes in the combined gVCF files were identified with GATK GenotypeGVCFs. The variant calls from GenotypeGVCFs for both haploid and diploid samples were postprocessed with the “vcfallelicprimitives” script and “vt normalize” function to change the representation of multinucleotide polymorphisms to SNPs (Tan *et al.* 2015). The haploid samples and diploid samples were genotyped separately with correct options for the ploidy of the samples. The variant call pipeline is available at https://doi.org/10.5281/zenodo.4750143.

### Genome-wide association analysis with 10-5 OP progeny

Sites with more than two alleles in the 10-5 OP progeny or with more than 20% missing data were removed, and the genotypes were converted to 012 coding with vcftools. The kinship matrix was calculated with the A.mat function from the rrBLUP package. The kinship matrix, genotype matrix, and phenotypes for the OP progeny were all input to the GWAS function in the rrBLUP package, with the additional settings of “fixed=NULL, min. MAF = 0.05, P3D=TRUE” (Endelman 2011). To visualize the results, scaffolds were assigned chromosome identifiers based on their assignment in the 12-linkage group map produced by Westbrook *et al.* (2015), with scaffolds not placed in a linkage group assigned to a separate linkage group (“NP”), and novel NLR transcripts not assigned to scaffolds were placed in a separate linkage group (“Novel NLR”) for data presentation purposes. Manhattan plot and qqplots were generated with qqman (Turner 2014).

Variants significantly associated with resistance to SC20-21 (*P*-value < 2.84 × 10^−6^) were selected for further annotation to evaluate (1) the segregation of the variant through allele frequencies in the 10-5 megagametophyte samples and the 10-5 OP progeny, (2) the predicted impact of the variant on any coding sequence, and (3) the location of the variant in the predicted protein in any coding sequence. Allele frequencies of the megagametophyte samples and the 10-5 OP progeny were obtained with vcftools (Danecek *et al.* 2011). The predicted impact of variants on the annotated gene products was obtained with vep (McLaren *et al.* 2010, 2016). Protein domains were identified with Interproscan 5 (Jones *et al.* 2014; Mitchell *et al.* 2019).

### Linkage map construction in 10-5

SNPs heterozygous in the maternal parent 10-5 were identified by their 1:1 segregation in the 32 10-5 megagametophyte samples using a chi-squared test (*P*-value ≥ 0.001). This allowed for SNPs with observed segregation distortion of up to about 3:1 to be considered heterozygous in 10-5. For linkage mapping, SNPs that were distorted beyond this level (*P*-value < 0.001) were removed prior to analysis.

A genetic linkage map of the 10-5 using the haploid population of 32 megagametophytes was calculated using Joinmap 4.1 with minimum logarithm of the odds (LOD) score set to 5.0, the regression mapping algorithm, and Kosambi’s mapping function. Only the first two rounds of mapping results were considered. The name of all linkage groups were dictated by the consensus linkage map from Westbrook *et al.* (2015).

### Quantitative trait locus mapping

To obtain a data set for quantitative trait locus (QTL) mapping in the OP family 10-5, we selected three types of SNP markers that were heterozygous in 10-5. Type 1 markers were those that produce only two genotypes in the progeny. These could be inferred as true testcross markers (10-5 or the maternal allele in the progeny is unambiguous) since the apparent minor allele frequency (MAF) in the pollen (male parents) is 0. Type 2 markers were those with three genotypes in the progeny and a MAF of ≤ 0.33 (and an estimated MAF in the pollen of < 0.15). For Type 2 markers, the maternal allele in the heterozygous progeny was inferred to be the minor allele. This produces a random error in <5% of the heterozygous scored progeny. Type 3 markers were those with three genotypes in the progeny and a MAF of > 0.45 (and an estimated MAF in the pollen parents of > 0.40). For Type 3 markers, the heterozygous progeny were re-coded to “missing” as the maternal allele cannot be reasonably predicted.

Composite interval mapping was performed using PLABQTL version 1.2bic (Utz *et al.* 1996). The phenotypic and SNP marker data of 291 half-sib samples were used for QTL analysis. Since PLABQTL was not originally designed to handle cross pollinated population data, we adapted the marker data from JoinMap format to double haploid data format in PLABQTL with the justification that only allele information from the mother tree was used. The linkage map from 32 megagametophytes was used in QTL analysis. The LOD threshold of 4.02 for QTL detection was determined by PLABQTL using a genome-wise error rate of 0.25. The genome wide scanning for the significant QTLs were performed for each 1 cM window. Finally, the effects of the detected QTL were further estimated by the “final simultaneous fit” procedure (simultaneous multiple regression using all detected QTLs).

### Association with previously identified *Fr1* candidate genes

Previous studies identified four markers linked to *Fr1* or candidate *Fr1* genes that have been localized in the *P*. *taeda* reference genome assemblies (Wilcox *et al.* 1996; Neale *et al.* 2014; Quesada *et al.* 2014; Amerson *et al.* 2015). Three out of the four *Fr1* candidate genes were included in this study’s hybridization probe set (Supplementary Table S2).

## Results

### NLR transcripts identified by de novo RNAseq assembly

The pooled RNA Illumina sequencing libraries resulted in an average of 26M (150 bp PE) reads per library, of which 24M were retained after QC. After assembly with Trinity, the libraries averaged 129,378 transcripts with an average of 103,115 open-reading frames per library identified by Transdecoder (Supplementary Table S3). Libraries from single families and libraries made with RNA pooled from multiple families did not differ significantly in the number of transcripts assembled, ORFs predicted, or NLR transcripts annotated (Supplementary Figure S2). On average, 22% and 35% of the annotated NLR transcripts from each library could be aligned to the Pita v1.01 and v2.01 reference genome assemblies, respectively, using 96% sequence identity threshold (Supplementary Figure S3). The NLR genes may represent sequence that is present in the genome of tree 20-1010 but was not assembled because of the inherent difficulty of assembling large gene families, or they may be sequences not found in the genome of tree 20-1010 (Weatherly *et al.* 2009; Warren *et al.* 2015; Clavijo *et al.* 2017; Alonge *et al.* 2020).

**Figure 2:**
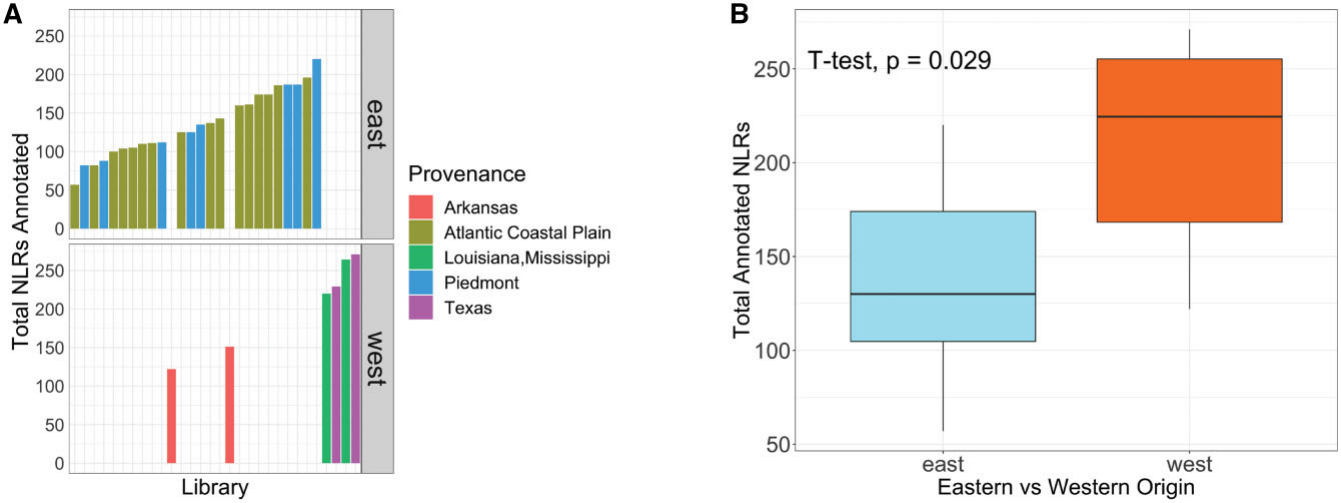
Number of annotated NLR transcripts. In (A), the number of NLRs annotated in each library is presented with bars colored by the provenance of the samples in that library (Arkansas in red, Atlantic Coastal Plain in gold, Louisiana/Mississippi in green, Piedmont in blue, Texas in purple). In (B), the distribution of the number of NLRs annotated in libraries of eastern (blue) *vs* western (orange) origin is presented. Plots were generated by ggplot2 (Wickham 2016).

NLRTracker annotated between 57 and 271 NLR transcripts in each of the denovo RNAseq assemblies of commercial *P.* *taeda* families (Figure  2A). The libraries with the most annotated NLR transcripts originated in TX and LA/MS. When grouped by eastern *vs* western seed sources (TX, AR, and LA/MS *vs* PDMT and ACP), the libraries of western origin have significantly more annotated NLR transcripts than the libraries of eastern origin (Figure  2B; Student’s *t*-test, *P*-value < 0.05). The number of annotated NLRs did not differ significantly between PDMT and ACP libraries (Student’s t-test, *P*-value < 0.05).

### Domain architectures of predicted NLR proteins

**Figure 3:**
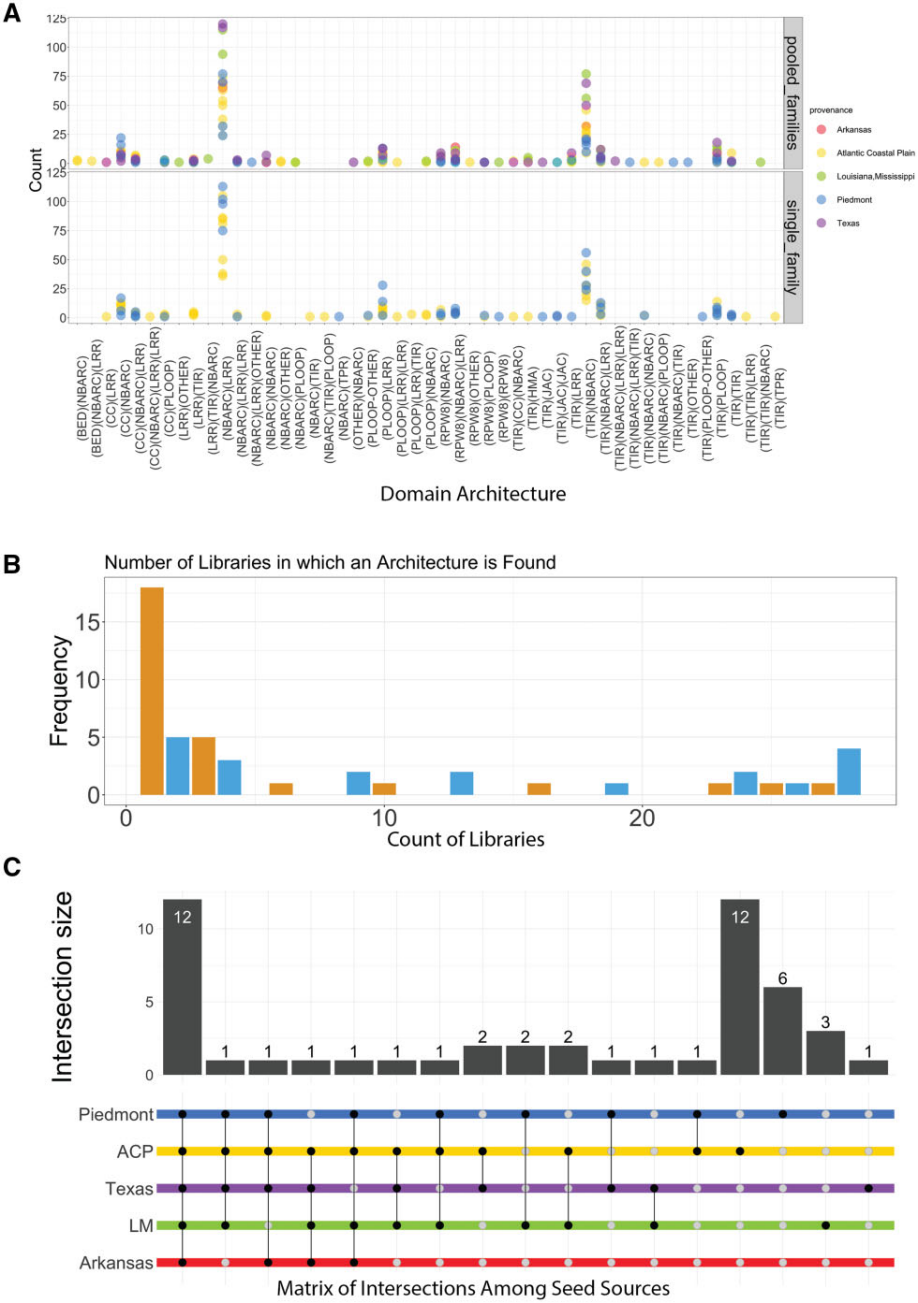
Private *vs* Shared Domain Architectures. (A) The count of NLRs in each domain architecture found in each library is shown with libraries with multiple families from the same provenance in the upper panel and libraries with a single family in the lower panel. (B) Histogram of the number of libraries in which each NLR domain architecture is found. In (C), an upset plot (Lex *et al.* 2014) is presented to visualize the overlap of NLR domain architectures between the provenances. The matrix below the bar chart indicates the seed sources included or excluded from each intersection. Empty sets are omitted from the upset plot. The bar chart indicates the number of NLR architectures in each intersection. Plots were made with ggplot2 (Wickham 2016) and ComplexUpset (Krassowski 2020). Colors in 3A and 3C are mapped to provenance as in Figure  2A.

In the 30 libraries, 49 different domain architectures were identified by NLRtracker (Figure  3A). Among the most frequent domain architectures identified are ones that suggest transcripts were sequenced and/or assembled incompletely [*i.e.*, (TIR)(NBARC) and (NBARC)(LRR)]. More unusual domain architectures were also found such as the “(BED)(NBARC)” and “(BED)(NBARC)(LRR)” and “(TIR)(CC)(NBARC)” architectures. Tallying the number of libraries in which a particular architecture is found, identified a set of seven core architectures found in more than 25 out of the 30 libraries, along with a larger set of 18 private architectures found in a single library (Figure  3B). When grouped by seed source, sets of domain architectures found only in libraries from particular seed sources were identified, with the ACP and PDMT seed sources having 12 and 6 private domain architectures, respectively; the LA/MS seed source having three private domain architectures; the TX seed source having one private domain architecture and the AR seed source having no private domain architectures (Figure  3C). When compared to domain architectures identified in gene models annotated in the Pita v2.01 genome assembly, 22 domain architectures were unique to our de novo assemblies (Supplementary Figure S4).

### Sequence-capture probe design and variant calling

Because many of the probes overlapped on the reference genome, the targets are described in terms of nonoverlapping regions. The probe set targeted 5984 nonoverlapping regions on 3229 genomic scaffolds. From the 30 elite rust-resistant family transcriptome assemblies, 1199 predicted NLR genes were targeted with one nonoverlapping region (one probe) each. Among the transcripts assembled, 2295 were annotated as NLR genes based on similarity to two or more of the expected NLR gene domains (either NB-ARC or TIR domains in conjunction with an NBS domain and/or LRR domain). The targeted regions covered a total of 2.9 Mbp of genomic sequence in the Pita v2.01 assembly. The targeted regions overlap 1232 annotated genes in the Pita v1.01 genome and 1199 predicted NLR genes from the elite resistant families’ transcriptomes.

### Genome-wide association analysis for resistance to SC20-21

**Figure 4:**
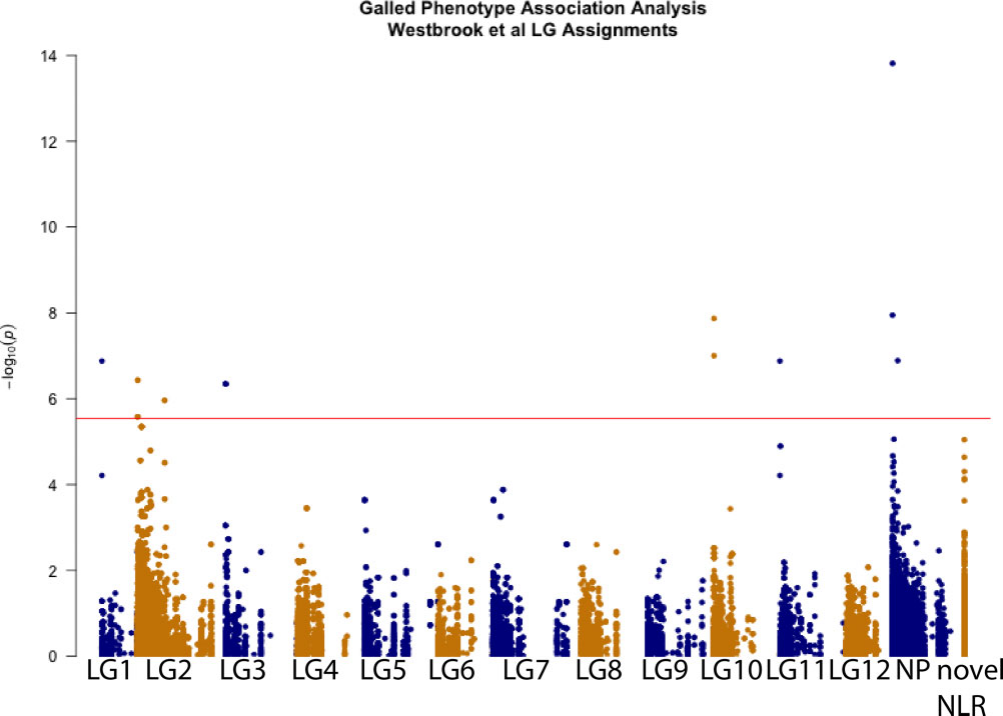
GWAS in open pollinated progeny of 10-5. Red line indicates a Bonferroni adjusted significant *P*-value of 2.84 × 10^−6^. SNPs are grouped and colored by the linkage group the scaffold was mapped to in Westbrook *et al.* SNPs on scaffolds not placed in a linkage group are colored in blue at right (NP). SNPs on novel NLRs included in sequence capture are colored orange at far right (novel NLR). The significant SNP in LG1 and LG11 is on a scaffold placed in both LG1 and LG11 in the Westbrook al. linkage map and is presented twice.

We analyzed SNPs associated with resistance to Cqf isolate SC20-21 in open pollinated 10-5 progeny with the mixed model rrBLUP and identified 10 significant SNPs (*P*-value 2.84 < 10^−6^; Figure  4). Three of the SNPs associated with resistance to SC20-21 were located on scaffolds placed in LG2 of the reference linkage map (Westbrook *et al.* 2015; Figure  4, Supplementary Table S4). Two of the SNPs on scaffolds placed in LG2 had a cM position and one did not. The two SNPs with a cM position on LG2 define an interval from 59.0 cM to 66.5 cM on LG2 in the reference map. One SNP associated with resistance to SC20-21 is located on scaffold C3901919 in the Pita v2.01 genome. The probes mapping to this scaffold were designed to target scaffold1961 in the Pita v1.01 genome; this scaffold was mapped to both LG1 and LG11 in the reference map. The remaining SNPs were located on scaffolds placed in LG3, LG10, or were on scaffolds not placed on linkage groups in the reference map. No significant GWAS SNPs were located on the novel NLR transcripts included in sequence capture.

Functional annotation of the GWAS significant SNPs in the Pita v2.01 and Pita v1.01 genome annotations did not identify any predicted impact on NLR genes (Supplementary Table S6). The sample of 20-1010 was heterozygous for 2 out of the 10 GWAS significant SNPs. The six 4-6664 × 10-5 trees were either homozygous or heterozygous for the same two GWAS significant SNPs as were one ACP sample and one PD sample that were genotyped (Supplementary Table S5).

### Linkage-map construction

Linkage map construction from the genotypes of the 32 megagametophyte samples resulted in 807 SNP markers placed in 27 linkage groups. The total length of this map is 1396 cM (Supplementary File S1). The largest linkage group, designated G1, contained mostly markers on scaffolds that had been placed in LG2 of the reference linkage map (Supplementary Figure S5). This same linkage group includes 3 out of the 10 significant GWAS SNPs as well as a scaffold that is the location of a prior *Fr1* candidate gene (“scaffold55875,” see Supplementary File 1 and Table S2). The three significant GWAS hits define an interval on G1, from 31.6 cM to 62.1 cM, with the scaffold55875 located at 62.1 cM.

### QTL analysis

QTL analysis with an additive gene model detected two QTLs: one on G1/LG2 (G1 in the 10-5 LG and LG2 in the reference linkage map) and one on G14 (which contains three scaffolds in the reference LG8, one placed in LG2 and LG8 and 24 scaffolds not placed in the reference map) (Table  2, Supplementary Figure S5). The QTL on G1/LG2 at 3 cM has a high partial *R*^2^ of 43.05%, indicating it is a major effect QTL. The QTL on G14 has a relatively low but still significant partial *R*^2^ of 4.75%. The overall adjusted *R*^2^ explains 43.6% of the phenotypic variance in the population.

**Table 2:** QTLs detected for resistance to SC20-21

G/Pos/Ref LG	CI (cM)	Cofactor	LOD	Part *R*^2^ (%)	Std Add
1/31/LG2	30–32	C3484301:1858	13.3	43.1	0.9
14/39/LG8	38–40	scaffold157211:746	4.4	4.8	0.2

Note: G/Pos/Ref LG, 10-5 linkage group/QTL position (cM)/reference linkage group; CI, support interval with 1 LOD fall off the peak, c. 95% confidence interval (cM); Cofactor, the SNP best representing the QTL effect; LOD, logarithm of the odds; Part *R*^2^ (%), percentage of phenotypic variance explained by one QTL when other QTL effects are fixed; std Add, additive QTL effect divided by the SD of the trait value. G/Pos, CI, Cofactor, and LOD were obtained from genome wide scanning; partial *R*^2^ (%) and std Add were further adjusted by the “final simultaneous fit” procedure.

## Discussion

### The first pan-NLRome for a gymnosperm species

The size and complexity of the *P*. *taeda* genome presents challenges for genome assembly and annotation (Kovach *et al.* 2010; Neale *et al.* 2014; Wegrzyn *et al.* 2014; Zimin *et al.* 2017). The current 2.01 version of the *P*. *taeda* reference genome from genotype 20-1010 consists of 1,489,469 scaffolds with a total length of 22 Gb. Based on loblolly pine and other conifer reference genomes, NLR gene families are large and contain many duplicated genes (Scott *et al.* 2020; Neale *et al.* 2014; Wegrzyn *et al.* 2014; Jiao and Schneeberger 2020; Stam *et al.* 2019). We hypothesized that widely planted elite rust-resistant families may possess family-specific NLR genes not identified in the *P*. *taeda* reference genome’s current draft assembly.

The NLR transcripts identified in our data are a first step toward a pan-NLRome in a gymnosperm species. *Pinus* *taeda* is an ideal gymnosperm species for a pan-NLRome study with its large natural geographic range. Prior NLR annotation efforts identified NLR transcripts in de novo-assembled transcriptomes from multiple conifer species or as part of annotating a single species’ genome. A recent study published long-read PacBio transcriptomes for two families of ACP origin (Lauer and Isik 2021). Our study is the first to explore the diversity of NLR transcripts across the geographic range of a gymnosperm.

Our results show some of the limitations of identifying NLR-encoding transcripts in transcriptomes assembled from short-read data, including a number of transcripts with domain architectures that suggest a truncated or incompletely assembled NLR transcript and that the number of NLR transcripts identified in a single library is substantially less than the number of potential NLR transcripts found in the Pita v1.01 and Pita v2.01 genomes.

To date, there is no comprehensive transcript support for the number of predicted NLR genes in the loblolly pine genome. Our identification of NLR transcripts in transcriptomes provide evidence for expression in young shoots, the tissue most susceptible to infection by the fusiform rust pathogen. Even with these limitations, we identified 22 domain architectures not found in the Pita v2.01 genome annotations, and a set of NLR transcripts not found in the Pita v2.01 genome assembly. The pooling strategy employed here to query more families did not significantly change the number of transcripts assembled and annotated in the pooled *vs* single-family libraries. This is in contrast to other pan-NLRome studies that identified NLR genes in genomes using long-read technologies (Witek *et al.* 2016; Giolai *et al.* 2017; Xing *et al.* 2018; Van de Weyer *et al.* 2019). Of particular interest are transcripts annotated as having a BED-NBARC and BED-NBARC-LRR domain architecture, which were found in two libraries from ACP seed sources. This domain architecture was previously reported to be exclusive to monocots and requires further investigation to confirm (Bailey *et al.* 2018; Marchal *et al.* 2018; Kourelis *et al.* 2020).

In addition to identifying NLR transcripts not found in the reference genome, we identified patterns of variation in domain architectures between the seed sources sampled in our study. While libraries prepared from families of western seed source had significantly more annotated NLR transcripts than libraries prepared from families of eastern seed source, the libraries prepared from eastern families had distinct architectures not found in libraries prepared from western families. These domain architectures include the BED-NBARC and BED-NBARC-LRR domain architectures. This aligns with previous research that identified a divide between *P*. *taeda* populations to the east and west of the Mississippi River (Ledig 1998; Schmidtling 1999; Al-Rabab’ah and Williams 2002; Schmidtling and Myszewski 2003; González-Martínez *et al.* 2006, 2007; Xu *et al.* 2008; Eckert *et al.* 2010). The cause of the difference observed in our study may be geographically heterogeneous patterns of purifying selection in ancient populations of *P. taeda*. However, we cannot rule out that some of these differences arose from recent artificial selection by 20th century breeding programs.

Past efforts to discover and annotate R genes in complex genomes (RenSeq; Jupe *et al.* 2013, 2014; Witek *et al.* 2016) designed baits from a reference genome to enrich for NLR genes in resistant samples. In contrast, our approach started with *de novo* transcriptomes from a broad array of samples of highly resistant families to discover novel NLR transcripts. The novel NLR transcripts were then targeted with hybridization probes for SNP identification. This study is the first application of a RenSeq-like approach in a gymnosperm (Jupe *et al.* 2013; Witek *et al.* 2016). Similar to RenSeq, our hybridization approach may be improved with long-read technology, which would aid in both mapping novel NLR genes to reference genomes and in SNP discovery and mapping. The set of probes targeting these putative NLR genes is a valuable resource for further studies targeting R genes in *P*. *taeda* and other pine species (Amerson *et al.* 2015).

We inferred that high rust resistance breeding values implied the presence of multiple, perhaps family-specific, R-genes. Through the generation of *de novo* transcriptome assemblies of rust-resistant families, we identified over a 1000 putative R genes. The linkage mapping analysis of 32 megagametophyte samples from the 10-5 maternal parent placed 35 out of the 1199 novel NLR transcripts in linkage groups. Because few of these NLR genes could be aligned to the Pita v1.01 or Pita v2.01 genome assemblies, we hypothesize that the unaligned sequences represent novel NLR genes.

### R gene linkage and discovery of SNPs through targeted genotyping of a single family

For mapping, we phenotyped and genotyped progeny from family 10-5 because the family is known to segregate for *Fr1.* The capture probes for genotyping used in this study targeted *Fr1* through two complementary approaches. First, by targeting LG2, we focused on detecting signals of *Fr1*-linkage in a genomic region previously shown to contain *Fr1*. Second, by using a large portion of probes to target novel NLR genes identified in the transcriptomes of rust resistant families, we attempted to detect signals of *Fr1*-linkage in candidate genes not present in the Pita v2.01 reference genome. The goal was to identify markers and their associated protein-coding genes that could be used to accelerate the development of rust resistant pines (Isik *et al.* 2008, 2012; Nelson *et al.* 2010; McKeand 2019).

Family based genome-wide association analysis identified 10 SNPs significantly (*P*-value < 2.84 ×10^−6^) associated with gall formation in 10-5 seedlings inoculated with basidiospores from Cqf isolate SC20-21. In a linkage map built from haploid megagametophyte (haploid maternal) samples, three of the significant SNPs were located on a single linkage group. This linkage group contained SNPs located on scaffolds placed in LG2 in prior genetic maps as well the scaffold with a candidate gene from a prior Fr1 mapping study (Neale *et al.* 2014).

We did not expect to identify the two SNPs on a scaffold mapped to LG10, and therefore unlinked to *Fr1*. This was unexpected, because in 10-5, the *Fr1* gene was the only *Fr* gene previously identified (Wilcox *et al.* 1996; Amerson *et al.* 2015). We cannot exclude the possibility that this is a technical artifact of an error in map location assignment of the SNPs to the scaffold, or the scaffold to the genome, since this gene family and the loblolly pine genome are both very complex. However, if the positioning is correct, this raises the possibility that the increased complexity of OP families (compared to full-sib families) might be leveraged to identify additional *Fr* genes derived from pollen parents, to which the inoculum is avirulent. This is supported by the fact that six samples from the 10-5 × 4-6664 cross had the reference (not GWAS significant) allele for several of the GWAS significant SNPs, while the four samples from commercial families that were genotyped possessed the alternate (GWAS significant) allele. Interestingly, LG2 contains *Fr1*, *Fr6*, *Fr7*, *Fr9*, whereas LG10 contains *Fr8*, all of which are R genes to which the single spore isolate SC20-21 is avirulent (Amerson *et al.* 2015). While 10-5 is known only to harbor *Fr1*, the OP family we screened may have additional *Fr* alleles introduced via the pollen cloud of rust resistant parents, which suggests our association analysis may have identified multiple *Fr* candidate genes in a single experiment.

### Leveraging southern pine and fusiform rust genetic resources

Our interest in fusiform rust is driven by the importance of breeding slash pine (*Pinus elliottii*) and loblolly pine (*P.* *taeda*) families called “elite rust-resistant families”; (Powers and Zobel 1978; Stelzer *et al.* 1999) for improved resistance to fusiform rust, decreasing the estimated $134M in annual losses caused by the disease (Cubbage *et al.* 2000). Genetic resistance to fusiform rust significantly reduces or eliminates the need for fungicide treatments in seedling nurseries, and leads to improved economic returns to land owners (McKeand *et al.* 2003; Walker and McKeand 2017; McKeand 2019). Given the persistence of the pathogen threat, fusiform rust disease resistance has always been a high priority for tree improvement efforts in the southern United States (McKeand *et al.* 2003).

Pine breeding programs provide abundant and highly informative germplasm for unraveling complex host–pathogen interactions at the molecular level. One example is the availability of improved tree genotypes, which we used to identify a set of disease R genes from the transcriptomes of families resistant to fusiform rust infection. Another resource is the availability of pedigreed materials—we analyzed an OP family to discover candidate *Fr* genes that can be used to accelerate breeding of disease resistant pines and guide the deployment of genetic resources. Since the repeating stage of the fusiform rust pathogen occurs on the oak host, and not the pine host, this approach should improve the expression of genetic resistance under field conditions. If we have successfully identified *Fr* genes(s) in this experiment, then this raises the possibility that we have identified markers for use in breeding programs to guide mating designs, to inform genetic selection, and to guide seedling deployment on the landscape where *Cronartium quercuum* pathotypes virulent to the corresponding *Fr* genes(s) are infrequent.

## Data availability

All fastq files generated in this study are available on SRA under BioProject accession PRJNA671612. The snakemake pipeline used for the variant-calling is available at https://doi.org/10.5281/zenodo.5762704. The assembled transcriptomes, annotated CDS sequences and translated amino acid sequences as well as a fasta file with the genomic scaffolds and novel NLR genes targeted by the hybridization probes and a bed file with the regions targeted by the hybridization probes are available at https://doi.org/10.5281/zenodo.5762704. Genotype and phenotype data are available at https://doi.org/10.5281/zenodo.5762704. Supplementary File S1 contains the linkage map generated from the 10-5 megagametophyte data. Supplementary material is available online at figshare: https://doi.org/10.25387/g3.17036426.
